# Potential natural polymer‐based nanofibres for the development of facemasks in countering viral outbreaks

**DOI:** 10.1002/app.50658

**Published:** 2021-03-09

**Authors:** Vigneshwaran Shanmugam, Karthik Babu, Thomas F. Garrison, Antonio J. Capezza, Richard T. Olsson, Seeram Ramakrishna, Mikael S. Hedenqvist, Shuvra Singha, Mattia Bartoli, Mauro Giorcelli, Gabriel Sas, Michael Försth, Oisik Das, Ágoston Restás, Filippo Berto

**Affiliations:** ^1^ Faculty of Mechanical Engineering Saveetha School of Engineering, Saveetha Institute of Medical and Technical Sciences Chennai Tamil Nadu India; ^2^ Department of Mechanical Engineering Centurion University of Technology and Management Sitapur Odisha India; ^3^ Chemistry Department King Fahd University of Petroleum & Minerals Dhahran Saudi Arabia; ^4^ Department of Fibre and Polymer Technology, Polymeric Materials Division School of Engineering Sciences in Chemistry, Biotechnology and Health, KTH Royal Institute of Technology Sweden; ^5^ Department of Plant Breeding, Faculty of Landscape Architecture Horticulture and Crop Production Science, SLU Swedish University of Agricultural Sciences Alnarp Sweden; ^6^ Department of Mechanical Engineering, Faculty of Engineering Center for Nanofibres and Nanotechnology Singapore Singapore; ^7^ Department of applied science and technology (DISAT) Politecnico di Torino Torino Italy; ^8^ Department of applied science and technology (DISAT) Istituto Italiano di Tecnologia (IIT) Torino Italy; ^9^ Structural and Fire Engineering Division, Department of Civil, Environmental and Natural Resources Engineering Luleå University of Technology Luleå Sweden; ^10^ Department of Fire Protection and Rescue Control National University of Public Service Budapest Hungary; ^11^ Department of Mechanical Engineering Norwegian University of Science and Technology Trondheim Norway

**Keywords:** biodegradable, electrospinning, fibers

## Abstract

The global coronavirus disease 2019 (COVID‐19) pandemic has rapidly increased the demand for facemasks as a measure to reduce the rapid spread of the pathogen. Throughout the pandemic, some countries such as Italy had a monthly demand of ca. 90 million facemasks. Domestic mask manufacturers are capable of manufacturing 8 million masks each week, although the demand was 40 million per week during March 2020. This dramatic increase has contributed to a spike in the generation of facemask waste. Facemasks are often manufactured with synthetic materials that are non‐biodegradable, and their increased usage and improper disposal are raising environmental concerns. Consequently, there is a strong interest for developing biodegradable facemasks made with for example, renewable nanofibres. A range of natural polymer‐based nanofibres has been studied for their potential to be used in air filter applications. This review article examines potential natural polymer‐based nanofibres along with their filtration and antimicrobial capabilities for developing biodegradable facemask that will promote a cleaner production.

## Graphical abstract

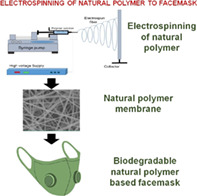

## 1 INTRODUCTION

### 1.1 Coronavirus and N95 facemasks

Elevated use of facemasks during the coronavirus disease 2019 (COVID‐19) pandemic has raised serious environmental concerns caused by the synthetic plastics used in protective facemasks, including the prevalent N95 type masks. It is estimated that about 3.5 billion N95 facemasks are needed in the United States alone during the pandemic.^1^ This dramatically increased the demand and consequently the production of N95 masks all over the world. For instance, 3M, the largest manufacturer of N95, has doubled its production rate by 1.1 billion per year from January 2020. Similarly, another manufacturer, Honeywell, also increased its production by 20 million N95 masks per month. 3M, Prestige Ameritech, and Honeywell are some of the notable manufacturers of facemasks. China produced 200 million facemasks per day in March 2020, which is 20 times higher than that produced in February 2020. The substantial rise in the use of N95 facemasks requires proper management and disposal methods to avoid adverse effects on the environment, human health, and safety, while preventing the possibility of a second wave of the epidemic.

N95 type of facemask is designed to filter airborne particles, with an efficiency of filtering 95% of ≥0.3 μm airborne particles according to National Institute for Occupational Safety and Health (NIOSH) N95 air filtration classification.^2^ NIOSH classified facemasks into three types, namely –N series, −R series, and –P series based on their resistance to oil and particle filtering efficiency when exposed to oil‐based aerosols, such as lubricants, cutting fluids, and glycerine.^3^ The 'N' type facemasks are non‐resistant to oil, 'R' type is moderately resistant to oil and the 'P' type is strongly resistant to oil or is oil proof. Each series is further categorized into three types based on their particle filtering efficiency, designated by '95,' '99' and '100,' which are N95, N99, N100, R95, R99, R100, P95, P99, and P100. The corresponding numeric value in the classification represents the filtration efficiency, that is, 95 represents filtration efficiency not less than 95%, 99 represents filtration efficiency not less than 99% and 100 represents filtration efficiency not less than 99.97%.^4^

N95 type facemasks are widely used in industries such as construction and mining for filtering dust and tiny air borne particles. However, special grades of N95 masks are recommended for health care applications to prevent the transmission of microorganisms and other particulate matter between health care professionals and patients. N95 masks can filter microorganisms such as bacteria and viruses, for example, Zhou et al.^5^ reported that the N95 type face mask had a filtration efficiency of 99.7% against influenza A virus, rhinovirus 14, and *Staphylococcus aureus*. National Personal Protective Technology Laboratory in NIOSH, which is part of the Centre for Disease Control and Prevention (CDC), sets standards and regulations for industrial type facemasks. For clinical type N95 facemasks, Food and Drug Administration‐USA standards and regulations are established under 21 CFR 878.4040 and CDC NIOSH under 42 CFR Part 84. N95 type facemasks are similar to those recommended by the European Union (FFP2 Respirators) and China Respirators (KN95) but there are variations in standard and performance.^6^ Other available facemasks with similar standards have also been used worldwide with approximately 94%–95% filtration efficiency, such as P2 (Australia/New Zealand), KP95 and KN95 (China), P2 (Brazil), FFP2 (Europe), DS2 and DL2 (Japan), BIS P2 (India), and 1st class such as the KF94 (Korean). The standards for these facemasks were structured by national regulatory standards, such as Australia/New Zealand Standard 1716, China GB2626, Europe EN 149, Japan JMHLW Notification 299, and Korea KMEOL 2017‐64. Table 1 shows the recommended filtration efficiency of different facemasks according to the national standard regulation.

**TABLE 1:** Filtration efficiency of different standard facemasks recommended by national standard regulation

Facemask type (standard)	Filter efficiency (must be ≥X%)
N95 (NIOSH‐42CFR84)	≥ 95%
FFP2 (EN 149‐2001)	≥ 94%
KN95 (GB2626‐2006)	≥ 95%
P2 (AS/NZ 1716:2012)	≥ 94%
Korea 1st class (Korea KMEOL 2017‐64)	≥ 94%
DS2 (Japan JMHLW Notification 214, 2018)	≥ 95%

N95 facemasks can minimize the airborne transmission of infective microorganisms by filtering out up to 95% of the particles from the air. During the rapid spread of COVID pandemic, a sudden spike in the demand for facemasks has resulted in their massive production of all types (one‐time use to long‐term use), majority of which are manufactured from nonbiodegradable synthetic plastics. Hence, it can be expected that casual disposal of these masks can result in serious pollution of the land and water bodies, adding to the already existing problem of plastic pollution. Furthermore, United Nations Conference on Trade and Development is urging governments all over the world to promote the use of non‐toxic, biodegradable and recyclable alternatives to reduce the mass production and consumption of synthetic plastics. In this scenario, developing biodegradable facemasks with effective microbial filtration capacity can not only alleviate the issue of plastic pollution but can also ensure safe health of individuals and easy disposal of masks. The main component of a facemask is the air filter, which is generally made of nonwoven synthetic plastic nanofibres such as polypropylene fiber, produced by the spunbonding and melt blown processes, which serve to remove particulates from the air. If these synthetic plastic nanofibres are replaced with natural polymer based nanofibres and biodegradable polymer fibers, it can reduce the use of plastic to a great extent, thereby minimizing the burden on the environment and promoting a cleaner production. The filter medium has two different structures‐woven type and nonwoven type. Woven type is used for filtering micron sized particles, whereas the nonwoven type is used to filter nanoparticles. The electrospinning process, since the last two decades, has been widely utilized to produce micro/nanofibres from biopolymers, which is an otherwise challenging task. The technique enables fabrication of nonwoven nanofibre membranes from natural biopolymers that can be used for air filtration applications.

### 1.2 Biodegradable natural polymers

Increasing industrial development around the world leads to the generation of huge amounts of plastic waste that causes serious environmental problems. Polymer membranes are of prime focus in this regard because of their extensive use in a variety of applications, such as packaging, gas separation, reverse osmosis, ultra, micro and nano‐filtration, and dialysis. Owing to their advantageous properties like high mechanical strength, microbial filtration efficiency, acceptable thermal properties, resistance to corrosion and chemicals, and tuneable chemical functionality. In the wake of increased environmental awareness, natural biopolymer‐based films and membranes have been used in the recent years for various aforementioned applications but most extensively in biomedical sector for tissue engineering, packaging and microbial filtration applications. Very recently, since the outbreak of COVID‐19, biopolymer‐based membranes have been acknowledged for use in facemasks due to their resistance to microbes and air filtration efficiency when electrospun into fibers.^7^, ^8^ Some notable natural biopolymers used for the production of bio‐based materials are polyesters, cellulose, chitin, chitosan, starch, and protein.^9^, ^10^, ^11^, ^12^, ^13^ Shortcomings in the synthetic polymer can be overcome by natural biopolymers, particularly due to their biodegradability and biocompatibility. When electrospun into nanofibres, they display efficient filtration performance and microbial resistance that justify them as suitable bio‐based air filtration alternatives to the existing filters derived from petroleum‐based synthetic polymers. Natural biopolymers have also been blended with synthetic polymers to enhance their performance.^14^ Various techniques, such as grafting, nanoparticle reinforcement, polymer blending and the use of customized copolymers, have been introduced and investigated in order to improve the performance of the membrane. Polymers, such as polyvinyl alcohol (PVA), polypropylene (PP), poly(glycolic acid), and poly (lactic acid) (PLA) have also been used in the development of polymeric membranes, which possess good mechanical strength and are also eco‐friendly in terms of biocompatibility and recyclability.^8^ Polymer membrane varies with pore nature and size with pore size being an important parameter for the classification of polymer membranes. Depending on the pore size, the membranes have been used in various applications. For microbial filtration, membranes with pore sizes of 0.1–5 μm are used for filtering particles such as bacteria and protozoa, and for filtration of ultra‐fine particles, such as proteins, viruses, colloids, and emulsified oils. Membranes with pore sizes of 0.01 and 0.1 μm are referred to as ultrafiltration membranes, and those with pore sizes ranging from 1 to 10 nm are referred to as nano filter membranes, that are used to remove nanoparticles. Furthermore, reverse osmosis membranes can filter particles ranging from 0.1 to 1 nm.^15^ Techniques, such as electrospinning, phase inversion, interfacial polymerization and casting technologies, are used in the processing and development of permeable/porous polymer membranes.^16^ Among these methods, electrospinning is the most popular that is cost‐effective and user‐friendly‐to‐develop into micro/nanofibre membranes of varying fiber diameter and pore sizes.

## 2 METHODOLOGY

This review article primarily focussed on the different available natural polymers, which could be electrospun to develop nanofibres for air filtration application in facemasks. Therefore, this review article will act as 'one‐stop‐shop' for researchers to identify potential natural polymers that has the potential to create sustainable filter medium in facemasks. The article also explains the state‐of‐the‐art of facemask production in the current scenario of the coronavirus pandemic and introduces the technique of electrospinning that can be used to manufacture nanofibre‐filtering mats.

The current review article was prepared by independently collecting relevant data from numerous scientific articles in various sub‐sections that were amalgamated in a rational manner to exclude information out of the scope for this review. All of the information, figures and tables reported in this article were provided with credits by citing and/or procuring permission from the publisher, wherever applicable. The articles used in this review were searched from the following databases: Science Direct, Google Scholar, PubMed, and Web of Science. The keywords used to search these articles were natural polymers, biopolymer, air filtration, facemask, and electrospinning. During the online search of articles, it was endeavored to restrict the time of publication for the past 20 years, however, in some cases, dated articles were cited since those were the fundamental studies. Based on the aforementioned criteria, ca. 150 articles were screened that contained relevant information needed for the development of the current article. These articles were then thoroughly studied to comprehend past research and to formulate a potential pathway for future research. Owing to the dearth of studies related to the electrospinning of natural polymers, less focus was on the number of citations or reputation of the publisher or the impact factor of the journal of the reviewed article. For this reason, in developing this review, the authors did not have the luxury to be overtly selective. However, based on the keywords mentioned before, interesting studies were identified and explained that would otherwise have remained obscured from the academic public eyes. In writing the sections 4 and 5 of this manuscript, the authors of the articles who focused their attention to electrospin various natural polymers and increasing their filtering efficiency and mechanical properties were incorporated into the current study. Overall, the review article is based on consistent and novel findings from a myriad of research available in the public domain, which was utilized to construct a narrative towards the need for facemasks made with sustainable materials that uphold the concept of cleaner production.

## 3 ELECTROSPINNING PROCESS AND NANOFIBRES

Electrospinning is widely acknowledged as an effective method used to produce biopolymer‐based nanofibres of diameter ranging from 2 to 500 nm.^17^ This technique is versatile because a variety of polymers can be fabricated into continuous nanofibres and is user‐friendly and inexpensive compared to other nanofibre processing methods. The other advantages of the technique include tuneable physical properties of fibers, ease of fiber functionalization, material combination, deposition on other substrates, and mass production capability. It is widely used in various applications, notably in biomedical sector for nano‐scale processing of tissue fibers. The electrospun nanofibres possess remarkable characteristics such as increased surface area to volume ratio and numerous micro‐sized pores.^18^ Because of these characteristics, electrospun nanofibres have been found to be attractive for bio‐medical applications. The nanofibres are also an effective reinforcing component in the preparation of polymer nanocomposites. The pore size can be varied during the electrospinning process, which makes them feasible for air‐filtration applications.

**FIGURE 1:**
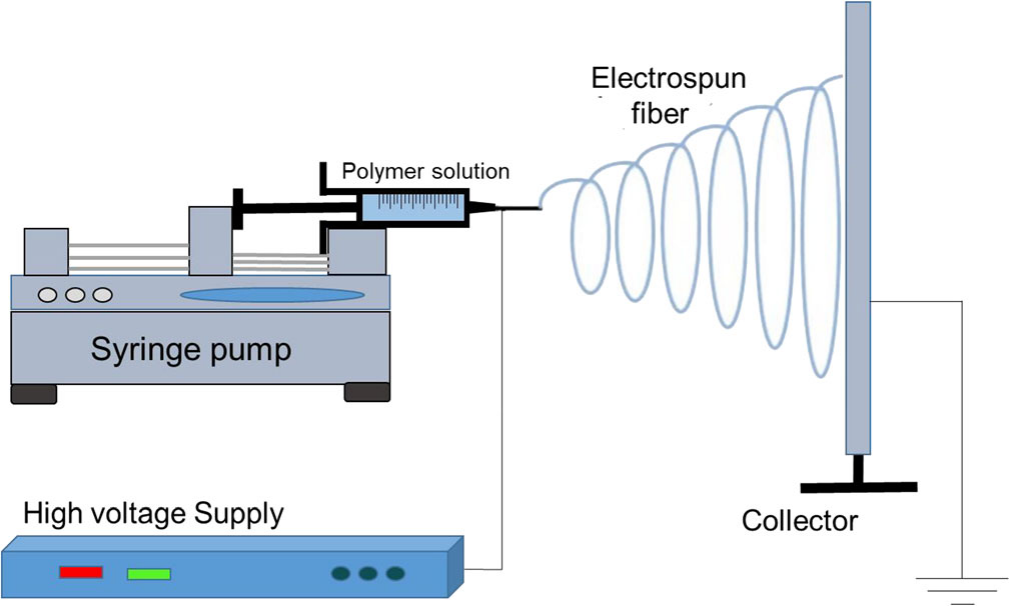
Schematic diagram of electrospinning process [Color figure can be viewed at wileyonlinelibrary.com]

Electrospinning method relies on an electric field to produce nanofibre at appropriate polymer concentrations. Figure 1 shows the schematic diagram of the electrospinning process. The electric field is applied to the polymer melt to create charge imbalance. The electric charge introduced creates tension in the melt and leads to the development of charged jet at the tip of the cone. The charging of the liquid polymer solution makes it ready for jet production. The charged jet is quickly focused on the target, where the polymer solution elongates, evaporates, and is produced as nanofibres. The solid nanofibres are thus deposited onto the target.^19^ Several factors have to be considered in the electrospinning process along with the polymer characteristics. It is necessary to consider the electrospinning parameters, such as the feed rate of polymer, the distance between the tip, and the collector, the electrical voltage. The surface tension and viscosity of the polymer solution play a crucial role in determining the concentration for steady electrospinning.^20^ While structure formation takes place at a millisecond rate, the degree of crystallinity and fiber crystal perfection are similar to those found in thicker fibers obtained from melt extrusion.^21^ Natural polymer nanofibres are difficult to manufacture through electrospinning process.^22^ As a result, there are only a handful of research articles that have reported natural polymer‐based electrospun nanofibres. However, it is important to understand that the natural polymer‐based electrospun nanofibre membranes have excellent filtration properties, which can be prudently used as air filters in facemasks.

**FIGURE 2:**
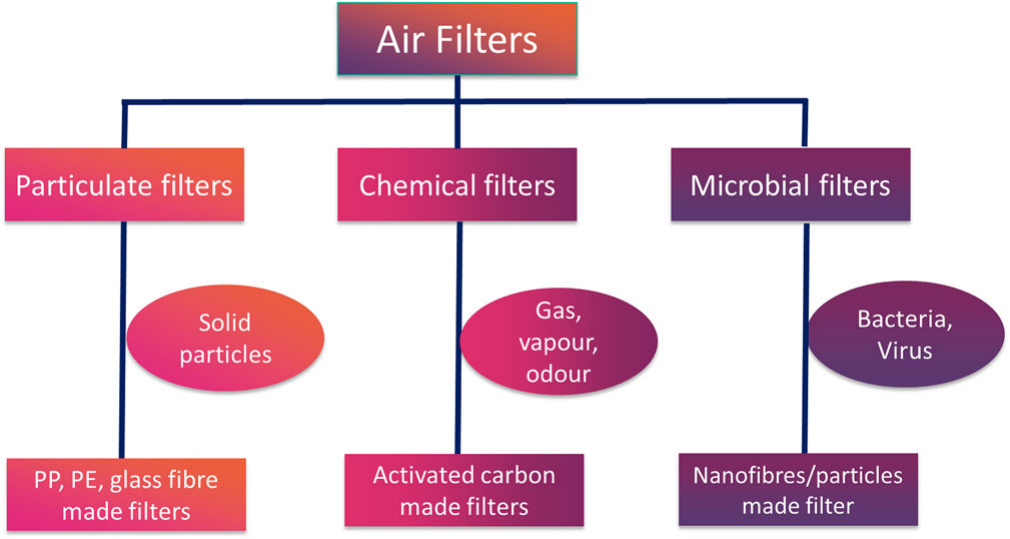
Types of air filters^31^ [Color figure can be viewed at wileyonlinelibrary.com]

Nanofibre membrane prepared with bio‐based polyesters has been reported to filter particles less than 500 nm owing to the improved specific surface areas/volume ratios.^23^ The highly porous nonwoven structure of fibrous electrospun membranes makes them suitable for microbial filtration, ultrafiltration, and nanoparticle filtration.^24^ Electrospun membranes can be bestowed with a high specific surface area and a porosity of 80% with both open and interconnected pores.^25^ It is to be kept in mind that porosity can be controlled by changing the diameter of a fiber by altering the electrospinning time. However, owing to the low interfiber interaction and weak adherence the membranes show poor mechanical properties. The very thin fibers are weak because of their very small cross section. Another major drawback in the electrospinning process is the issue of bio‐fouling. Such shortcomings can be addressed by using nanomaterials as a reinforcement agent, appropriate surface chemistry modifications and/or by blending with other high‐strength bio‐based polymers.^26^, ^27^ Qin and Wang, 2008 ^28^ proposed the development of multilayer electrospun membranes with cross‐linked nanofibres to increase the air filtration efficiency. The electrospun membranes' filtration efficiency can be managed by optimizing the pore size. By changing the operating parameter of the melt and the electrospinning conditions, nanofibres of varying diameter and pore sizes can be achieved.^29^ Incorporation of cellulose nanocrystals in the cellulose acetate membrane formed hierarchical structures that increased the membrane's filtration and mechanical properties and hydrophilicity.^30^ The different types of air filters are illustrated in Figure 2.

## 4 BIODEGRADABLE NATURAL POLYMER BASED NANOFIBRES

### 4.1 Cellulose

Cellulose nanofibres (CNF) or CNF filaments produced from wood material are known to have remarkable properties, such as good consistency, lightweight, durability, and transparency. The CNF fibers are about 2–20 nm in width and 0.5–2 μm in length.^32^ Cellulose is a hydrophilic polysaccharide that makes up about 40%–90% of the composition of plants. Cellulose crystallinity ranges from 65% to 95% based on the source origin.^33^ There are six crystalline structures that have been identified: I, II, III1, III2, IV1, and IV2 cellulose. Among these, the cellulose I is the most preferred material because it is abundantly available in nature.^34^ A single cellulose fiber consists of several micro fibrils that are linked together by strong hydrogen bonds, which lead to its improved physical characteristics. The hydrogen bonding enables the strong cellulose to cellulose interaction, which improve the interlocking properties of the fiber.^35^ CNFs in plants play an important role in maintaining the strength and integrity of the cell wall through a hierarchical organization. They are the strongest component and the largest load‐bearing structures in trees and plants. To separate nano fibrils from the cell wall, several energy effective methods were implemented.^36^ The CNF is profoundly feasible for load bearing applications because of its high mechanical strength. CNFs come in different forms, such as cellulose whiskers, micro fibrillated fibers, electrospun nanofibres, and bacterial cellulose nanofibres.^37^ The diameter of the electrospun CNF, bacterial cellulose and micro fibrillated cellulose is 50–500, 2–4, and 10–100 nm, respectively. All of these differ in their form, size and morphology based on the plant source.^38^ The CNFs are extracted from various natural plant sources, some of which are curaua fiber,^39^ sisal fiber,^40^ banana peel,^41^ cotton,^42^ corn cob residue,^43^ wheat straw,^44^ and pine fiber.^45^ Nevertheless, it has been challenging to achieve completely disintegrated CNFs without causing any damage to the cellulose structure, owing to the complicated existence of the cell walls and the strong interfiber hydrogen bonding.

Liu and Hsieh^46^ have successfully developed ultrafine cellulose membranes with uniform diameters (100 nm to 1 μm) by electrospinning cellulose acetate using different collectors. Four different types of collectors were used, namely paper, aluminum foil, copper mesh, and a water‐based collector. The membrane developed on copper mesh and paper collector was found to be highly porous than the membrane developed on aluminum foil and water‐based collector. The water retention of the developed cellulose membrane was 10 times higher than that of the commodity fibers produced fabrics. It should be noted that cellulose‐based materials are hydrophilic and thus need to be addressed in order to be used as facemask material. Chattopadhyay et al.^47^ investigated the ability of cellulose‐based electrospun membranes to filter aerosols. The results of filtration were compared with commercial glass fiber filters with an average fiber diameter of 0.5 μm and cellulose acetate microfiber material with an average fiber diameter of 24 μm. The electrospun cellulose membrane showed better quality factors at a lower membrane thickness (7–43 μm) compared to the glass fiber membrane. When the layer thickness was increased above 40 μm, the particle penetration had a negligible variation, however, the increase in the pressure drop reduced the quality factor. The author did not report the filtering efficiency of the developed membrane corresponding to the quality factor. Matulevicius et al.^48^ compared the filtration efficiency of electrospun polyamide, polyvinyl acetate, polyacrylonitrile and cellulose acetate nanofibre media for aerosol filtration. The filtration efficiency of the cellulose acetate membrane was lower than 91%, even at its highest quality factor. However, a maximum filtering efficiency of ca. 99% (filtering 100 nm particles) and ca. 97% (filtering 300 nm particles) was reported for polyvinyl acetate‐based filter media. All of these investigations have strong recommendations for the use of cellulose in face mask filter media, although cellulose has poor water resistance properties that need to be addressed.

### 4.2 Chitin

Chitin is the second most prevalent polysaccharide naturally available biopolymer after cellulose that is, mostly found in shellfish and insect exoskeletons, mollusks, and mushroom cell walls. Chitin nanofibres have a diameter of ca. 2–5 nm and a length of ca. 300 nm enclosed in a protein matrix.^49^ It is estimated that nearly 10^10^–10^11^ tons of chitin is biosynthesized every year.^50^ While chitin is a semi crystalline natural biopolymer with fibrillar morphology of nano size diameter and excellent material properties, most chitin is discarded as industrial waste without effective utilization.^50^, ^51^ Annual shellfish manufacturing firms produce 75% of crustacean by‐products that is, discarded as waste and the lack of effective waste management strategies leads to potentially severe environmental hazards.^52^ It is therefore essential to promote efficient use of chitin as a green material, which is environmentally friendly. Chitin is semi crystalline with strong interchain hydrogen bonding due to its linear structure with two hydroxyl groups and an acetamide ring.^53^ Chitin polymer chains are arranged in an antiparallel conformation in the chitin nanofibres.^54^ The strong hydrogen bonds are responsible for the fibrillar morphology and make the structure stable.^55^ It has a few special properties that make it attractive in the biomedical field, such as biocompatibility, good mechanical strength, acceptable thermal stability, and chemical resistance.^56^, ^57^ However, insolubility of chitin in most common organic solvents has restricted its application to a great extent. Moreover, owing to the strong hydrogen bonding it is difficult to isolate single fibers from a bunch of fibrils. Various approaches have been implemented to obtain consistent chitin nanofibres, such as the use of grinders, TEMPO‐mediated oxidation, and partial deacetylation.

Electrospinning of chitin has not been extensively investigated because it is insoluble in water and common organic solvents.^50^ However, Ji et al.^58^ used chitin to develop electrospun nanocomposite mat based on chitin‐nanofibril/polycaprolactone. The nanocomposite was electrospun at varying mass ratios of chitin nanofibril and polycaprolactone (5:95, 10:90, 15:85, 20:80, 25:75, and 30:70). The addition of chitin increased the mechanical strength and modulus of the nanocomposite. Nanocomposite with 5:95 ratio of chitin/polycaprolactone showed a yield stress of 13 MPa but it was increased to 21 MPa for 30:70 nanocomposite. The highest modulus of 500 MPa was observed for 20:80 nanocomposite, which was 150% higher than the pure polycaprolactone.

Similarly, in another study, electrospun chitin and polycaprolactone composite mat exhibited superior toughness and melting enthalpy to that of pure polycaprolactone, which was 1852% and 180%, respectively.^59^ Furthermore, in the smoke filter test, chitin and polycaprolactone composite mat showed 350% superiority in particulate matter (2.5 μm in size) filtering compared to the pure polycaprolactone mat. These results demonstrate the effectiveness of the use of chitin as a reinforcement material for electrospun membranes.

### 4.3 Chitosan

Chitosan is a polysaccharide acquired by *N*‐deacetylation of chitin and it is made of glucosamine and *N*‐acetylglucosamine units.^60^ Owing to its availability, biodegradability, biocompatibility, and binding energy it has been extensively researched for different applications. They are also used in biomedical applications due to antibacterial properties. Alkaline treatment procedures have been adopted to extract chitosan from chitin, such as the NaOH hydrolysis that contributes to *N*‐deacetylation of chitin. Chitosan is soluble only in dilute aqueous acidic solution (pH < 6.5). However, alterations in chitosan's chemical structure, through chemical modifications, make them soluble in common organic solvents. Chitosan consists of regularly organized hydroxyl groups and amine linked by *D*‐glucosamine units.^61^ The presence of hydroxyl groups in chitosan makes them crystalline in solid state, however forms fibrils in solution with strong hydrogen bonding. This property supports its effective electrospinning and increases its use in many applications. However, chitosan has poor thermal and mechanical properties that can be improved by different methods. Abdul Khalil et al.^62^ addressed the properties of chitosan and stated that the strengthening of chitosan using cellulose fibers can increase the physical properties of the resulting material. Nanocomposites or fibers developed with chitosan have improved thermal and mechanical properties, however they are water sensitive.^63^

The poor solubility of chitosan in water makes it difficult to be used in the electrospinning process. However, by blending with other polymer, electrospinning of chitosan was successfully accomplished. Wang et al.^64^ developed ultrafine chitosan hybrid nanofibre (PVA, TiO2, and Ag nanoparticles) mat by electrospinning and investigated the characteristics of air filtration. Hybrid chitosan fibers were deposited on a nonwoven PP substrate to develop a filter membrane. The electrospun nanofibre was 25–60 nm in diameter. The increase in the diameter of nanofibre reduced the filtration efficiency due to the increased pore size at a larger diameter. The filtration efficiency was enhanced by increasing the thickness of the nanofibre layer. Maximum efficiency of 99.2% was noted for the membrane with a nanofibre layer thickness of 18.7 μm. In another study, Li et al.^65^ developed a PLA/chitosan based fiber composite membrane and investigated the filtration performance. During electrospinning, the chitosan and PLA mass ratios were varied as 0:8, 1:4, 1:8, 1.5:8, 2:8, and 2.5:8. The chitosan/PLA membrane with a mass ratio of 2.5:8 showed a maximum filtration efficiency of 98.99% with a pressure drop of ca. 148 Pa. The authors therefore suggested the PLA/chitosan‐based membrane for air filter media. Zang et al.^66^ investigated the in situ electrospun‐chitosan nanostructure filtration efficiency against 2.5 μm particle size. The filtration efficiency was compared to similar electrospun poly (acrylonitrile) PAN, poly (vinylpyrrolidone) (PVP) and polystyrene (PS) structures. The particle matter removal rate for chitosan nanostructure was ca. 4 μg m^−3^ s^−1^, while for PAN, PVP and PS structures it was approximately 2, 3 and 2 μg m^−3^ s^−1^, respectively. This result shows the efficacy of the in situ electrospun method for the development of chitosan‐based membranes for filtration applications.

### 4.4 Alginate

Alginate is a polysaccharide that is widely distributed in the cell walls of brown algae. Alginate fiber is derived by alkaline treatment of the polymer, followed by precipitation using calcium chloride. After the purification step, fibers are extracted/obtained as sodium alginate fiber.^67^ The composition in the alginate and alginate fibers differs from the source of brown algae. Alginate fibers are widely used in wound dressing applications due to their gelling properties, biocompatibility, biodegradability, and nontoxic nature. Electrospinning of alginate is difficult, because alginate tends to gel at high polymer concentrations when the solution becomes highly viscous. However, alginate can be electrospun by the introduction of copolymers.^68^ The authors introduced alginate to poly (ethylene oxide) polymer. Electrospun alginate/poly (ethylene oxide) was produced in two different combinations, that is, 70:30–80:20, and was cross‐linked with calcium chloride. The electrospun alginate/poly (ethylene oxide) nanofibre had a diameter of about 75 nm. Alginate/poly (ethylene oxide) nanofibre with a cross‐link of calcium chloride exhibited tensile modulus of 5 MPa and for non‐cross‐linked nanofibre it was 7 MPa. The investigation recommended electrospinning of alginate with different polymers in order to increase its use in biomedical applications. However, due to the gelling properties of alginate, it is not directly used in air filtration applications but can be mixed with other biopolymers and fabricated into air filter membranes. Venkatesan et al.^69^ developed an alginate and chitosan‐based membrane with silver nanoparticles for microbial filtration. Antimicrobial activity of the electrospun membrane was tested against *Escherichia coli* and *Staphylococcus aureus* microorganisms. A uniform dispersion of silver nanoparticles with a pore size of 50–500 μm on the electrospun membrane was observed. The addition of silver nanoparticles increased the bacterial filtering efficiency 1.5 times higher than the alginate‐chitosan membrane. This investigation emphasizes the use of silver nano‐particles in the polymer for filtering microorganisms. Further research could be extended in this area to find an optimized percentage of silver nano‐particles to achieve 99% bacterial filtering efficiency. In another study, Dodero et al.^70^ investigated electrospinning of alginate‐based mats with and without ZnO nanoparticles. The electrospun mats were cross‐linked with calcium, strontium and barium ions. The tensile strength and elongation of the mat were increased due to the cross‐linking. Maximum strength of 21 MPa was noted for the strontium cross‐linked mat and its corresponding elongation at break was 6%. The same mat showed a low moisture content of 11%, hence the author reported that the strontium cross‐linked mat was capable of being stored for a long time without having any negative effect on its properties.

### 4.5 Collagen

Collagen is a protein present in animal bones and tissues, such as cartilage, tendons, muscle, and skin. The size and shape of collagen vary from species to species. It constitutes 25% of total protein weight and 70%–80% of the total flesh weight of vertebrates and is plentiful in nature.^71^ The physical properties as well as the collagen composition depend on the processing method and characteristics of the enzymes used during extraction. Collagen fiber is typically 280 nm long with strong hydrogen and intermolecular bonds and consists of three helical polypeptide chains made of amino acids.^72^ Collagen is of different types and 29 forms of collagen have been identified and reported which differ in structure and fibril characteristics.^73^ The most common types of collagen are Type I and Type II. The Type I fibers are found in the connective tissues and the Type II fibers are found largely in the cartilage tissues. The Type III collagen is found in abundance after Type I and II collagen. The production of the Type III collagen varies with age: Young animal tissues contain about 50% of the Type III collagen, which gradually reduces to 5%–10% with age.^74^ Chemical and enzymatic hydrolysis are the two most common methods used for collagen extraction. Chemical hydrolysis is the preferred route of extraction in the industry due to low processing costs. However, high quality collagen fibers are extracted by enzymatic hydrolysis. Additionally, enzymatic hydrolysis yields low amount of waste by‐products compared to the chemical hydrolysis.^75^ The collagen fibers possess low mechanical strength, however, blending with synthetic polymers results in improved properties of collagen fibers. Collagen nanofibres obtained through electrospinning is extensively used in biomedical applications. Recently, Kim et al.^76^ developed cotton like collagen bundle through modified electrospinning process. Carlisle et al.^77^ investigated the mechanical bending stress–strain results of single electrospun collagen Type I nanofibres. The modulus of the collagen fiber decreased when the strain was increased that led to the strain softening of fiber. The average modulus reported for electrospun collagen Type I fibers was 2.8 GPa. Similar to this investigation, Yang et al.^78^ investigated the bending properties of single electrospun collagen Type I nanofibres. The bending modulus of nanofibre decreased from ca. 7.6 to ca. 1.4 GPa with an increase in nanofibre diameter up to ca. 250 nm. Due to the shearing between segments of electrospun collagen fibers, the nanofibre diameter showed a significant influence in the bending modulus. It is understood from the studies that it is critical to identify the properties of single collagen fibers that could contribute to the design of nanofibre mat with the desired properties for specific applications.

### 4.6 Gelatine

Gelatine is a protein derived from collagen by partial hydrolysis. During the partial hydrolysis of collagen, pretreatments and different extraction methods are followed at controlled hydrolysis conditions.^79^ Typically, rich fibrous gelatine is obtained from the bones and skin of the animals. Gelatine is an effective material for biomedical applications due to its biodegradability and biocompatibility.^80^ The main attributes of gelatine are its gel strength and thermal stability.^81^ The molecular properties, such as molecular weight distribution and amino acid composition aid in the determination of gelatine's gel strength and thermal stability.^82^ The quality and application depends on the physical properties of gelatine, which, in turn is related to the structure of the polymer chains.^83^ The choice of gelatine for a specific application is based on its rheological characteristics. Gelatine extraction at low temperature results in increased stiffness.^84^ Wang et al. developed gelatine based nanonets.^85^ The nano nets possessed spider‐web like structure owing to the existence of hydrogen bonds between the gelatine chains. Ki et al.^86^ successfully prepared gelatine nanofibres and studied electrospinning parameters affecting the spinning ability of gelatine and its morphology. The fiber diameter of the gelatine fibers was controlled by dope concentration during electrospinning. When increasing the concentration of the dope, a linear increase in the fiber diameter was noted. An average diameter of 169 nm was observed at a concentration of 12%. However, the fiber was found to be uniform and fine at a concentration of 8% with an average diameter of 86 nm. Huang et al.^22^ found increased mechanical strength of the electrospun nanofibre mat with a fine fiber structure. Gelatine/2,2,2‐trifluorothanol electrospun nanofibre was produced at varying mass concentrations of gelatine (2.5%, 5.0%, 7.5%, 10%, 12.5%, and 15%). It is interesting to note the maximum tensile strength of 4.5 MPa and a Young's modulus of 174 MPa were measured for 7.5% gelatine nanofibres with, which is 40%–60% higher than the other nanofibre produced. Zhang et al.^87^ studied the effect of cross‐linking on the mechanical properties of electrospun gelatine nanofibre. Saturated glutaraldehyde was used for cross‐linking. The cross‐linked gelatine nanofibre showed a tensile strength and a modulus 10 times higher than that of non‐cross‐linked gelatine nanofibre. Both studies have shown that the concentration of mass as well as the cross‐linking of gelatine is critical in increasing the mechanical strength of the gelatine nanofibre, which could offer increased strength when it is used for making air filter membrane.

### 4.7 Silk fibroin

Silk fibroin is a type of fibrous protein formed by arthropods, such as silkworms, bees, mites, scorpions, and spiders. During metamorphosis of these species, the protein is spun into fibers.^88^ The biological nature, composition, and properties of the silk vary depending on the source of extraction. However, the silk extracted from silkworms is the preferred silk material and is widely used in textile products. A silk cocoon produces 600–1500 m of fiber.^89^ *Bombyx mori* is the widely used silkworm species because of its excellent fiber properties and the ease of domestication of these worms.^90^ Silk fiber comprises of two kinds of proteins, (a) silk fibroin (approximately 75%) and (b) sericin (approximately 25%). Silk fibroin is the fibrous protein that is, insulated with sericin. Sericin is soluble and is removed during fibroin extraction.^91^ Silk fibroin possesses excellent mechanical strength and is biocompatible. The protein is semi‐crystalline in nature due to the strong interchain hydrogen bonding, which is the reason for the increased stiffness of these fibers.^92^ The molecular weight of the protein varies between 200 and 350 kDa.^85^ Based on the structure, silk fibroin is classified into three groups: Silk I, Silk II, and Silk III.^93^ Silk fibroin based nanofibre has been widely used in biomedical applications such as tissue engineering and wound dressing. Kim et al.^94^ developed silk fibroin nanofibres using electrospinning process. The nanofibre produced was reported to have an average diameter of 80 nm and the diameter distribution ranged from 30 to 120 nm. Recently, Kopp et al.^95^ optimized electrospinning parameters to produce silk‐based nanofibres without the use of any additives during electrospinning. Yin and Xiong,^96^ fabricated silk fibroin and polycaprolactone‐based composite nanofibre mats and analyzed their mechanical properties with respect to different fiber orientations. The distribution of fiber orientation affected the mechanical characteristics of the fiber mats. Amiraliyana et al.^97^ produced silk fibroin made electron spun nanofibre mat and examined their tensile strength and modulus. The strength of the nanofibre mat was higher for the smaller nanofibre mat diameter. Maximum tensile strength of ca. 19 MPa was noted for nanofibre mats with an average fiber diameter of 85.5 nm, whereas for fibers with a diameter of 165.3 and 206.8 nm it was ca.16 and ca.11 MPa, respectively. The lower strength of the thick nanofibre was due to its lower molecular orientation. Wang et al.^98^ recommended silk fibroin made nanofibre membrane as an efficient material for lightweight air filter media. The nanofibre mat was produced at three different electrospinning times, that is, 5, 10, 20 min. Silk fibroin made nanofibre membrane has a higher filtration efficiency than commercial microfiber membranes, namely PP fiber cloth, PP fiber cotton, KN90 respirator, and glass fiber membrane. The silk fibroin nanofibre membrane produced at 20 min of electrospinning displayed a filtration efficiency of 98.8% with a pressure drop of 98 Pa against particle matter of 2.5 μm (PM 2.5) size. In addition, the doping of silver nanoparticles to the nanofibre membrane bestowed effective resistance against *E.coli* and *S.aureus* microorganisms. Gao et al.^99^ reported similar results where silk fibroin/polyethylene‐oxide‐based nonwoven fibrous membrane has been developed through electrospinning. The silk fibroin nanofibre air filter showed an improved filtration efficiency of 99.99% for PM 2.5 that have higher quality factor. Overall, these investigations demonstrate that silk fibroin produced nanofibre membrane is an effective material for air filter media due to its high efficiency, low airflow resistance, light weight, biocompatibility, and multi‐functionality.

### 4.8 Keratin

Keratin is the abundant biocompatible and biodegradable protein found in the hairs, nails, horns, and wool of animals as well as in feathers of birds and in fish scales. Based on the sulfur content present as well as the physical and chemical properties, keratin is classified as soft keratin and hard keratin. The amino acid chains in keratin differ in number, size, charge polarity and the amino acid sequence.^100^ Typically, wool contains about 82% of keratin protein whereas chicken feather contains about 91%.^101^ However, the chemical composition and properties of the keratin varies with source. Keratin has excellent hydrophilicity and adsorption characteristics.^102^ Ma et al.^103^ reported that keratin can be fabricated into different forms, such as nanoparticles, nanofibres, scaffolds, films and can potentially be used in packaging/filtration applications, and in cosmetics. Keratin has the ability to absorb harmful compounds, such as formaldehyde, heavy metal ions, and other pernicious volatile organic compounds, which makes it an ideal candidate for filtration applications.^104^ It is also used in biomedical applications due to its fibrous structure and biocompatible nature. The appropriate method for fabricating keratin to a fibrous membrane without altering its properties is electrospinning.^105^ However, the production of keratin membranes is difficult due to its insolubility in water and organic solvents, low viscoelastic properties, and low molecular weight.^106^ Figoli et al.^107^ recommended membrane keratin for filtration applications. The study revealed filtration efficiency of the keratin membrane against microorganisms, heavy metals, and organic materials, as well as harmful gasses and air. He et al.^108^ developed keratin/poly (vinyl alcohol) composite nanofibres by electrospinning. Two different types of nanofibre were developed: random nanofibre and aligned nanofibre. Compared to random nanofibre, a maximum tensile strength of 11 MPa was noted for the aligned keratin/poly (vinyl alcohol) made nanofibre. From this investigation, potential options for increasing the strength of the keratin nanofibre membrane through fiber alignment were identified. Further investigation of a different fiber orientation could lead to interesting results, which would open up the possibility of developing high‐strength nanofibre keratin. Shen et al.^109^ reported the filtration efficiency of the silver nanoparticles doped with keratin/polyamide6 nanofibre membrane. Nanofibres have been developed with varying percentages of keratin (0%, 30%, 50%, and 70%). The 30% keratin nanofibre membrane showed a high filtration efficiency with a quality factor of 0.044. However, the increase in keratin reduced the efficiency of filtration due to the low pore size. The addition of silver nanoparticles to nanofibre increased the antimicrobial properties of nanofibre. The average bacterial filtration efficiency was 96.8% for *S.aureus* and 95.6% for *E.coli*. Overall, it is noted that keratin nanofibres are capable of exhibiting increased mechanical properties, air filtration characteristics and antimicrobial action. These properties make keratin nanofibre an effective candidate for face mask development, in which it can be used as an effective medium for air filters.

### 4.9 Prolamin‐based protein

These are a group of storage proteins, with high proline amino acid content, found mainly in the seeds of plant cereal grains, such as wheat (gluten), sorghum (kafirin), corn (zein), and barley (hordein). These proteins can be spun into nanofibres which are completely biodegradable.^110^, ^111^ Prolamin‐based protein is currently used in the food industry owing to the high glutamine and proline content. These protein‐based polymers are effective candidates with properties comparable to that of synthetic polymers. Recently Das et al.^112^ developed a framework for the development of gluten‐based facemasks. The recommended, 4 wt% lanosol treatment of gluten to enhance the fire retardant properties that resolve the issue of flammability in gluten based facemasks. Dong et al.^113^ developed gluten‐based nanofibres with poly (vinyl alcohol) and thiolate poly (vinyl alcohol) additives. Additives were added to the gluten at different ratios. The diameter of electrospun nanofibres varied with regard to the concentration and interactions between additives and gluten, however maximum diameter of 1225 nm was noted for gluten/PVA nanofibre at a ratio of 10:1. This means that it is important to prefer the optimized ratio of additives in the development of nanofibres with a defined diameter. Xiao et al.^114^ investigated the tensile properties of hybrid polycaprolactone (PCL) and kafirin‐based nanofibre mats developed by electrospinning at different mass ratios of kafirin/PCL (1:0, 3:1, 2:1, 1:1, 1:2, 1:3, and 0:1). Nanofibre mats developed at the 1:0, 3:1, and 2:1 ratios of kafirin/PCL were found to be rigid and brittle. Nanofibre mat having kafirin/PCL at a ratio of 1:2 showed maximum elongation, however, a maximum tensile strength of 6 MPa with a modulus of 4 MPa was observed for nanofibre mat having kafirin/PCL at a ratio of 1:3. From this investigation, it is identified that the increased mass ratio of kafirin enhances the brittleness of the composites, which could contribute to lower tensile strength.

Deng et al.^115^ developed electrospun zein/gelatine based nanofibre mat at different weight ratios of gelatine/zein (1:0, 3:1, 2:1, 1:1, 1:2, and 1:3). The nanofibre mat was thermally cross‐linked in an oven for 3 h at 140°C and their wettability and tensile properties were investigated. The cross‐linked nanofibre mat showed increased water resistance, notably the 1:3 ratio cross‐linked gelatine/zein mat showed a reduced weight loss of about ca. 13% with a swelling ratio of ca. 2 g/g. Compared to the pure gelatine nanofibre mat, gelatine/zein nanofibre mat showed lower weight loss due to increased water resistance offered by zein. However, the tensile strength and modulus of gelatine/zein nanofibre mat were compromised. The maximum elastic modulus of 78 MPa, the tensile strength of 2 MPa, and the elongation at break of 41% were observed for the cross‐linked gelatine fiber mat. Recently, Yu et al.^116^ developed electrospun zein‐based nanofibres by blending with polyvinyl alcohol. Nanofibres have been developed with a ratio of 60:40 (zein/polyvinyl alcohol) and cross‐linking with varying concentrations of glutaraldehyde (0%, 10%, 20%, 30%, and 40%). The cross‐linked nanofibre had a superior air filtration efficiency of between 92% and 98%. The 40% cross‐linked nanofibres displayed a filtration efficiency of 98% for pollutant particles greater than size 0.5 μm and a filtration efficiency of 97.3% for 0.3 μm sized particle. The cross‐linked nanofibre with 30% glutaraldehyde showed a maximum tensile strength of ca. 3 MPa, while the non‐cross‐linked nanofibre had a maximum tensile strength of ca. 1.5 MPa. Overall, it is understood that the cross‐linking of zein‐based nanofibre is effective for water resistance, air filtration and improved mechanical strength, making it viable for the development of facemask filter media.

## 5 IMPORTANT CONSIDERATIONS IN FACEMASK FILTERS

Over the years, air filtration membranes have been used to filter particulate matter. The filtration membranes play a predominant role during the filtration process and affect the filtration efficiency to a great extent. Recent developments in filtration technology have led to the emergence of several new materials with unique physical and chemical properties to improve filtration performance. However, electrospun membranes are inevitable for filtration application due to their high efficiency in filtering micro and nanoparticles. Assisted by the advancement of the electrospinning method, the application of electrospun nanofibre membranes has become ubiquitous in the field of air filtration. The main advantages of electrospun nanofibre membranes are their tuneable fiber characteristics, such as optimisable diameter, pore structure, and high surface area‐to‐volume ratio that make them superior to the conventional filtration membranes. According to the theory of filtration, the process involves two stages that is, stable stage and unstable stage. In the stable stage, flow resistance and filtration efficiency are unchangeable with respect to time, whereas in the unstable stage flow resistance and filtration efficiency change with respect to time and are independent of the particle properties.^117^ The filtration membrane is said to be efficient when it offers possible filtration in a variety of environments and conditions while ensuring the safety of human beings.^118^ Further, in most cases, non‐biodegradable and some toxic organic solvents are used to develop the nanofibre membranes. The use of biodegradable polymers for the development of nanofibre membranes for filtration applications is an effective solution. The use of green and biodegradable polymers is a good alternate solution that prevents the use of unsafe organic solvents that affect human health.^119^ As mentioned in Section 3, the various available natural polymers can be used to develop filtration membranes. In the recent years, several studies have reported the filtration performance of natural polymer‐based electrospun nanofibre membranes that demonstrated their potential to be used for filtration applications. Table 2 shows the key results found in the research of natural polymer‐based electrospun nanofibres. Zhu et al.^119^ reported that the filtration membrane developed with chitosan‐based natural biopolymer showed excellent air and microbial filtration, while the addition of silica nanoparticles to the membrane increased the roughness, which further enhanced the filtering efficiency. Ahne et al.^120^ reported 99.8% filtration efficiency of the electrospun cellulose‐based nanofibre. Electrospinning of cellulose acetate nanofibres over polypropylene nonwoven material improved the filtration performance of the material from 50% to 91%.^121^ Leong et al.^122^ suggested cellulose‐based nanofibre as a viable filtration medium over N95 masks. Soybean protein‐based nanofibre fabric was developed by Souzandeh et al.^123^ The fabric exhibited superior filtration performance for airborne particulates as well as toxic gaseous chemicals. Further study revealed the soybean protein‐based nanofibre fabric to be a highly efficient multifunctional air filter material. The bacteria filtering performance of soy bean protein‐based polymers was investigated by Lubasova et al.^124^ The filter material was developed by electrospinning the blend of soya bean protein and poly (ethylene oxide) and the properties were studied at different blend ratios of the two polymers. The blended medium had a 100% efficiency in filtering *E.coli* bacteria, while the pure poly (ethylene oxide) nanofibre displayed only 81.5% of bacteria filtering efficiency. Desai et al.^125^ developed chitosan and polyethylene oxide‐based filter media using the electrospinning process whereby the properties were studied as a function of varying chitosan fiber diameter and content. The investigation revealed that the size and content of the chitosan fibers were the dominant factors that influenced the filtration performance. Wang et al.^98^ compared the performance of silk nanofibre air filter membrane to the commercially available KN90 respirator as well as the polypropylene nanofibre membrane. The silk nanofibre membrane exhibited the same filtration performance as the commercially available filter membranes. The findings of the investigation suggested the use of silk nanofibre as a suitable alternative to the petroleum derived polypropylene for constructing the air filter medium. In another study, the silk fibroin based filter medium demonstrated a filtration efficiency of 99.99% for filtering particulate matter with size ranging from 0.3 to 10 μm.^99^ Some studies also reported the use of hybrid nanofibres in air filter applications in the recent years.^126^, ^127^ Developing a hybrid natural polymer‐based biodegradable filter medium may boost the filtering properties and also help to achieve the desired microbial resistance, water resistance and mechanical strength characteristics.^31^ These findings demonstrate the potential of the natural polymer‐based nanofibres for use in air filtration applications that also can be considered to construct the filter material for facemasks.

**TABLE 2:** Some basic details of electrospinning process and outcomes

Filtration materials	Application	Working parameters	Results	Ref.
Cellulose acetate and PP non‐woven	Fabricate multilayered filtration material	Needle diameter: 0.7 mm, f = 0.3 ml/h, d = 10 cm, and V = 25 kV.	The layer of nanofibres electrospun onto PP nonwoven material increased the *η*_f_ of PP nonwoven material from 50.23% to 91.29%, however, the *Q*_f_ reduced by 29.1%. When the cellulose acetate deposition time was increased from 3 to 6 h, the *η*_f_ further increased to 98.26% and the *Q*_f_ was relatively increased by 0.6%. The mean pore size was 0.463 and 15.640 mm for CA nanofibres and PP nonwoven material, respectively.	Omollo et al.^121^
(PVA)/cellulose nanocrystals (CNCs)	Fiber‐based filters for indoor air purification	A 5 ml syringe with a 22‐G needle, d = 10 cm and V = 22 kV.	The thinner fibers reduced pressure drop significantly and enhanced the efficiency of particulate matter removal. 99.1% of *η*_f_ was achieved in extremely polluted conditions (the mass concentration of particle diameters ≤2.5 μm is 500 μg m^−3^) with low pressure drop (91 Pa) at an airflow velocity of 0.2 m s^−1^.	Zhang et al.^128^
Chitosan / PEO	‐	f = 0.08 ml/min, d = 10 cm, and V = 30 kV	Increasing the fiber diameter, the *η*_f_ decreased because the maximum pore size and air permeability increased. With increasing fiber diameter, the polystyrene bead *η*_f_ decreased. This is likely due to higher maximum pore size observed with increasing fiber diameter along with increase in air permeability.	Desai et al.^125^
Chitosan Nanoparticle/PLA	Air filtration and antibacterial performance	A 5 ml syringe with a 21‐G needle tip, f = 1 ml/h, d = 14 cm, and V = 18 kV.	Compared to the pure PLA membrane (99.90%), the *η*_f_ of PLA/chitosan fibrous membranes was slightly lower at 98.10%–98.99%, whereas the chitosan content had almost no effect on the *η*_f_. However, the pressure drop of pure PLA membrane was 335.90 Pa and it decreased to 167.05 Pa when the mass ratio of chitosan to PLA was 1:8. When the mass ratio of chitosan to PLA was 2.5:8, the *Q*_f_ (up to 0.0312) was the highest, which indicated the best filtration performance.	Li et al.^65^
Silk protein nanofibres/PEO	Multifunctional air filters	The 21 G nozzle tip, d = 20 cm, f = 10 μL/min and V = 10 kV	Air η_f_ of the fabricated SNAFs could reach up to 90% and 97% for PMs with sizes under 2.5 and 10 μm, respectively, exceeding the performances of commercial semi‐high‐efficiency particulate air (semi‐HEPA) filters. After use, the SNAFs could be naturally degraded.	Min et al.^129^
Gelatin/*β*–cyclodextrin Bio–nanofibres	Respiratory filter media	The 23 G nozzle tip, f = 0.15 ml/h, d = 20 cm, and V = 22 kV	Gelatin/*β*‐cyclodextrin nanofibres captured aerosols (0.3–5 μm) with ˃95% *η*_f_ at 0.029/Pa *Q*_f_. They adsorbed significant amount of xylene (287 mg/g), benzene (242 mg/g), and formaldehyde (0.75 mg/g) volatile organic compounds.	Kadam et al.^130^
Ag doped keratin/PA6 nanofibre	Air filtration and antimicrobial performance	A 15 ml syringe with a 20 G flat‐tip needle, f = 0.1 ml/h, d = 25 cm, and V = 20 kV	The addition of the Ag nanoparticles (AgNPs) imparted a strong antibacterial activity to the composite membrane against *S. aureus* (99.62%) and *E. coli* (99.10%). Bacterial *η*_f_ of the composite membrane against *S. aureus* and *E. coli* were up to 96.8% and 95.6%, respectively. The usage of coarse wool in bio‐protective air filters could offer tremendous economic benefits to enterprises.	Shen et al.^109^
Keratin (K) –polysulfone (PS) blend	Wastewater treatment applications	A 5 ml syringe with a 22 G needle, f = 0.8 ml/h, d = 12 cm, and V = 12 kV.	The performance of PS‐K membranes in tannery effluent treatment resulted in 76% enhanced dye removal efficiency.	Karunanidhi et al.^131^

Abbreviations: d, tip to collector distance; f, feed rate; *Q*_f_, quality factor; *η*_f_, filtration efficiency; SNAF, silk nanofibrous air filters; G, gauge.

The filter used in facemasks should be resistant to the microbial organisms. The presence or passage of microorganisms in/through the filter can result in serious health hazards or can even be fatal. In order to counter such issues, antimicrobial nanoparticles can be incorporated in the membrane that can prevent the growth and transmission of microorganisms, thereby ensuring the safety of the wearer.^132^ Medical facemask materials should be tested in accordance with the available standard testing procedures and other specific requirements. The standard testing methods followed in the manufacturing of medical facial masks are shown in Table 3. According to the available standard testing methods and requirements, facemasks are tested for fluid resistance, filtration efficiency, differential pressure, flammability, microbial cleanliness, and biocompatibility. As per the ASTM F2100 specifications, the facemasks used in health centers are classified under three major levels based on their barrier protection ability and the specifications are shown in Table 4. Additionally, Oberg and Brosseau^133^ defined four key features for mask performance filter efficiency, moisture resistance, flammability, and differential pressure. In addition to all these, masks should also possess good mechanical properties.^112^ All these factors must be taken into consideration while designing a facemask. For instance, the methodology proposed by Das et al.,^112^ recommended gluten and gluten‐derived biochar‐based biodegradable facemasks with improved microbial filtration properties, good mechanical strength, as well as resistance to water and fire.

**TABLE 3:** Testing standard followed for medical facemasks

Standard	Description
ASTM F1862	Test method for resistance of medical facemasks to penetration by synthetic blood (horizontal projection of fixed volume at a known velocity)
ASTM F2101	Test method for evaluating the bacterial filtration efficiency (BFE) of medical facemask materials, using a biological aerosol of *Staphylococcus aureus*
ASTM 2100	Standard specification for performance of materials used in medical facemasks
ASTM F2299	Test method for determining the initial efficiency of materials used in medical facemasks to penetration by particulates using latex spheres
42 CFR Part 84	Approval of respiratory protective devices
29 CFR Part 1910.1030	Occupational exposure to blood‐borne pathogens: final rule
16 CFR Part 1610	Standard for the flammability of clothing textiles
BS EN 14683:2019	Medical facemasks. Requirements and test methods (British standard)
ISO 2859‐1:1999	Sampling procedures for inspection by attributes sampling schemes indexed by acceptance quality limit (AQL) for lot‐by‐lot inspection
ISO 10993‐5, 10	Test for skin sensitivity and cytotoxicity to ensure that no materials are harmful to the wearer.

**TABLE 4:** ASTM levels for facemasks^134^

Level	Fluid resistance (mm hg)	Differential pressure–breathability (mm H_2_O/cm^2^)	Microorganism filter efficiency	Sub‐micron particulate filtration efficiency (@ 0.1 micron)	Flammability (flame speed)
ASTM Level 1: low barrier protection	80	<4	≥95%	≥95%	Class 1 (≥3.5 s)
ASTM Level 2: moderate barrier protection	120	<5	≥98%	≥98%	Class 1 (≥3.5 s)
ASTM Level 3: maximum barrier protection	160	<5	≥98%	≥98%	Class 1 (≥3.5 s)

## 6 SUMMARY AND FUTURE SCOPES

**FIGURE 3:**
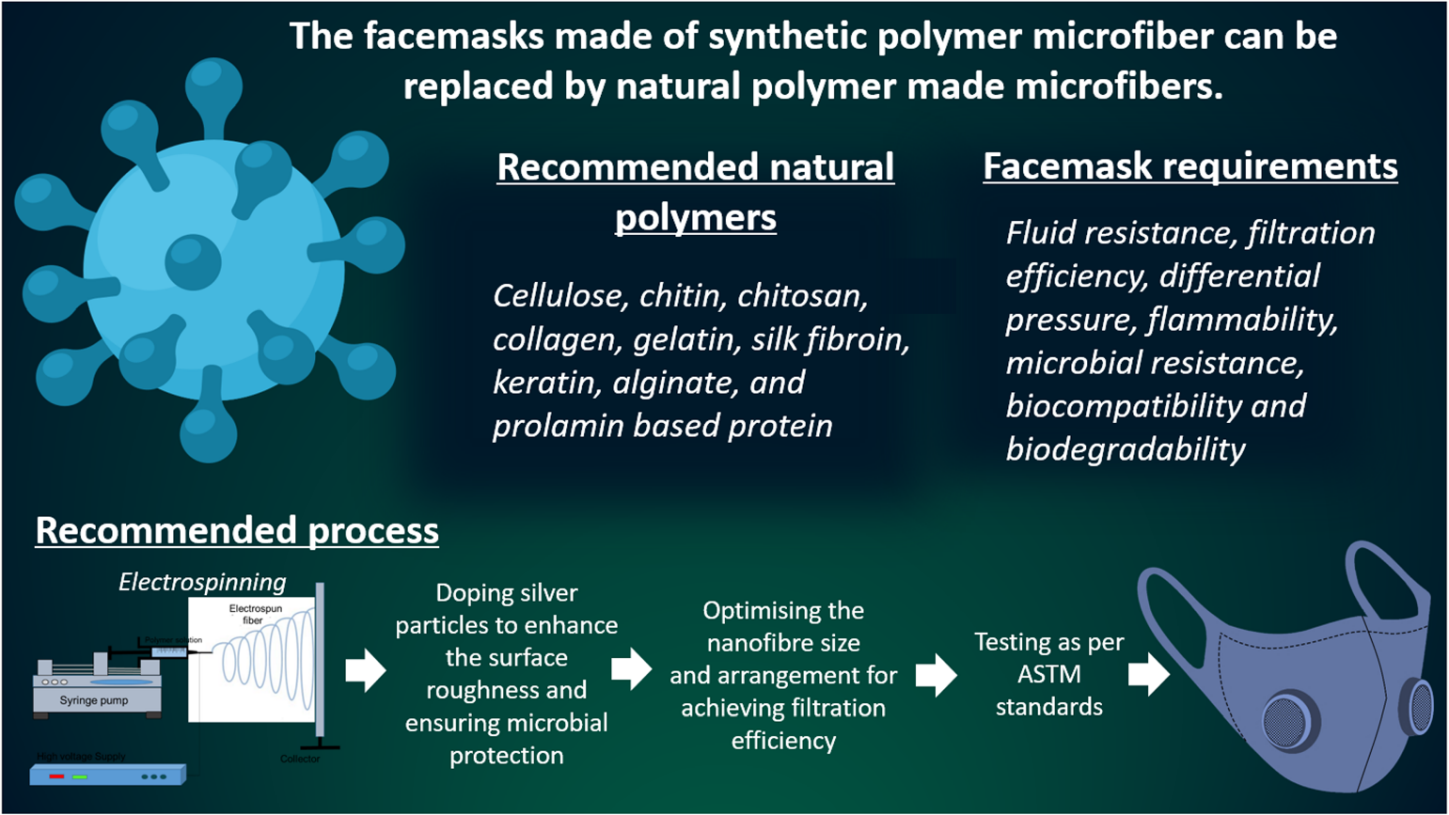
Biodegradable facemasks [Color figure can be viewed at wileyonlinelibrary.com]

The possibilities for developing biodegradable facemasks from natural polymers have been discussed in the present review. Polymers obtained from the natural sources are identified as effective candidates for use as filter media in facemasks. Natural polymers can be fabricated into nanofibres by electrospinning whereby the fiber length and diameter, and the pore size can be controlled. Through sensible optimization of the nanofibre dimensions, pore size and arrangement, natural polymer based nanofibres can be used to construct efficient air filters for facemasks, which can be non‐toxic, biodegradable and hence eco‐friendly alternatives to the existing petroleum‐based polymers. Furthermore, to enable antimicrobial properties, the fiber membranes can be doped with silver nanoparticles. The doped silver particles enhance the surface roughness and ensure microbial protection. Figure 3 shows the recommended natural polymers and processes for the development of biodegradable facemasks. Although the air filtration performance of natural polymer‐based nanofibres has been demonstrated through a number of investigations, research works focused on the development of natural polymer‐based mask filter is inadequate and requires an interdisciplinary approach whereby, a concerted effort by chemists, biologists and engineers is required. Successful development of biodegradable masks will foster a sustainable environment and cleaner production that reduce the harmful environmental effects of synthetic plastic masks. Research in this area can also encourage the use of biodegradable natural polymer in various other applications that would have a huge positive impact on the society and the environment.
